# A Novel Method for a COVID-19 Classification of Countries Based on an Intelligent Fuzzy Fractal Approach

**DOI:** 10.3390/healthcare9020196

**Published:** 2021-02-10

**Authors:** Oscar Castillo, Patricia Melin

**Affiliations:** Tijuana Institute of Technology, Tijuana 22414, Mexico; ocastillo@tectijuana.mx

**Keywords:** fractal dimension, fuzzy logic, classification, COVID-19

## Abstract

We outline in this article a hybrid intelligent fuzzy fractal approach for classification of countries based on a mixture of fractal theoretical concepts and fuzzy logic mathematical constructs. The mathematical definition of the fractal dimension provides a way to estimate the complexity of the non-linear dynamic behavior exhibited by the time series of the countries. Fuzzy logic offers a way to represent and handle the inherent uncertainty of the classification problem. The hybrid intelligent approach is composed of a fuzzy system formed by a set of fuzzy rules that uses the fractal dimensions of the data as inputs and produce as a final output the classification of countries. The hybrid approach calculations are based on the COVID-19 data of confirmed and death cases. The main contribution is the proposed hybrid approach composed of the fractal dimension definition and fuzzy logic concepts for achieving an accurate classification of countries based on the complexity of the COVID-19 time series data. Publicly available datasets of 11 countries have been the basis to construct the fuzzy system and 15 different countries were considered in the validation of the proposed classification approach. Simulation results show that a classification accuracy over 93% can be achieved, which can be considered good for this complex problem.

## 1. Introduction

We describe in this article a novel hybrid approach for the classification of countries based on the COVID-19 data complexity. The proposed approach consists of a hybridization of fractal theoretical constructs and fuzzy logic concepts to achieve the goal of classifying the countries based on the complexity of their time series data. The definition of the fractal dimension [1] is used to estimate the geometrical complexity of the data. There is a wide variety of algorithms for calculating the fractal dimension producing a crisp value by using as data the time series for a particular problem. This value offers an estimation of the complexity of a specific time series. Using the ranges of the crisp values for the fractal dimension estimations for different data, we can construct fuzzy values for the fractal dimensions and then the fuzzy rules with the ability to classify countries based on the COVID-19 data [2]. The rules represent the classification knowledge and can be elaborated with the help fuzzy clustering on the data [3]. The main idea is that fuzzy logic will model the inherent uncertainty in the classification decision process. The hybrid intelligent approach can be applied by performing the next steps. First, we outline the specific set of fuzzy if-then rules using the fractal dimension values as inputs. Then, we specify a method for estimating the fractal dimension. Finally, the numeric values of the fractal dimension are used as inputs to the set of fuzzy rules to perform the classification.

The fuzzy system can be established in the form of the Mamdani reasoning method, and centroid as the defuzzification approach [4]. However, it is also a viable option to use a Sugeno fuzzy model, where the consequents are mathematical expressions and usually are consider to be linear equations [5]. In this case, it is also possible to consider a neuro-fuzzy method for parameter adaptation in the fuzzy model based on real data of the problem. We can use, as one possible alternative, the adaptive neuro-fuzzy inference system approach (ANFIS) [6] to learn from real data the optimal coefficient parameter values of the linear equations and for the membership functions [7]. In this case, the proposed hybrid approach for COVID-19 time series classification was built with the Mamdani fuzzy method. The main reason for deciding for the Mamdani approach is because of the fact that is totally linguistic, which means that a Mamdani fuzzy model uses fuzzy inputs and outputs, and because of this it is more transparent for the experts in the area. On the other hand, the Sugeno approach involves equations in the outputs that are not so easy for experts and users to fully understand and trust.

In recent times we have become aware of the rapid spread of COVID-19 in all the world, reported initially in China and then spreading to nearby countries, and later to other continents. For the particular case of Europe, several countries like Italy, Spain, France, and Germany were hit very hard with the spread of COVID-19, with a lot of confirmed cases and deaths [8,9,10,11,12,13]. Later, in the American continent, the United States was also hit hard with the COVID-19 pandemic [14,15,16,17]. So, it is of utmost importance that vigorous research work should be done for comprehending all facets of this Pandemic problem [18,19,20]. In the case of this paper, we are focusing our attention on the classification facet, which means constructing groups of countries according to their similarities.

The main contribution of the paper is the hybrid approach composed of fuzzy logic and fractal dimension for the classification of countries based on the respective COVID-19 time series data. A fuzzy system is outlined, aimed at encapsulating the knowledge needed to classify the countries. The fractal dimension concept allows measuring the complex behavior of times series. Furthermore, since the COVID-19 data are of countries worldwide, we expect that the contribution will have a relevant and important impact and benefit for nations worldwide. The importance of achieving a classification of countries based on the COVID-19 data is that forecasting the time series and deciding possible control actions depend on current situation of the countries. In addition, the class of a particular country can change in time and this can be measured in different time windows as the fractal dimension changes accordingly. In addition, international agencies could use the global information of all countries to make international decisions to control the spread of the virus. On the other hand, the method could be also used at the level of regions inside a country to control the spread in the regions of a particular country.

The remaining sections of the paper are structured in the following way: Section 2 reviews the basic concepts for understanding the fractal dimension calculation. Section 3 explains the main concepts of applying fuzzy logic for classification. Section 4 outlines the hybrid intelligent approach for classification formed by a prudent combination of the fractal dimension to estimate complexity of the data and fuzzy logic for encapsulating the knowledge of classification. Section 5 describes the simulation results. Section 6 describes some possible applications of the proposed classification method. Finally, Section 7 outlines the main conclusions based on the obtained results and proposes possible future works for near, medium and long terms.

## 2. Theoretical Background on the Fractal Dimension

**Figure 1:**
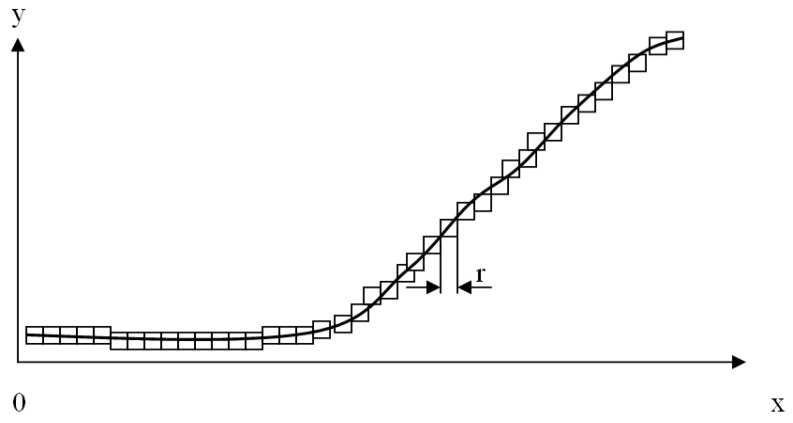
Illustration of the box counting algorithm for an arbitrary curve C.

In recent times, significant advances have been achieved in studying the geometrical complexity of objects with the help of fractal theory [1]. As an instance, financial and economical time series exhibits a fractal structure [21,22]. In fact, the fractal theory concepts have found interesting applications in medicine, food industry, robotics, and control. One of the definitions for the fractal dimension is the following one:

(1) $$d\underset{r\to 0}{=}\lim\left[\mathrm{lnN}\left(r\right)\right]/\left[\ln\left(1/r\right)\right]$$

where N(r) stands for the number of boxes covering a particular object and r stands for the size of the box. The estimation of the numeric value of the fractal dimension can be obtained by counting the number of boxes covering the object for different r values and then calculating a least squares regression to estimate the numeric value of d (box counting algorithm). In Figure 1, an illustration of the box counting algorithm for an arbitrary curve C is presented. Counting the number of boxes for different sizes of r and then calculating a regression, we can estimate the crisp value of the box dimension of an arbitrary geometrical object using the equation:

lnN(r) = lnβ − dlnr(2)

where d represents the fractal dimension, and a least squares method can be applied to estimate this crisp value based on the provided data.

The fractal dimension can be used as a general method to characterize any arbitrary object. The main reason for this statement is that the fractal dimension estimates the geometrical complexity of objects. For the case of this paper, a time series is classified by using the numeric value of the fractal dimension (the value of d is between 1 and 2 because data are on the xy plane). The main reasoning for this classification scheme is that when the boundary of the object is smooth then the fractal dimension value of the object will be near to one. On the other hand, when the boundary of the object is rougher the fractal dimension value will be near to a value of two.

## 3. Basic Concepts for a Classification Approach Based on Fuzzy Logic

Fuzzy logic provides the basic concepts to build a fuzzy system that can serve as a classification method by making an appropriate granulation of the input space such that we can distinguish among different geometrical objects by their essential characteristics. The proposed approach will be outlined on the plane for simplicity, but can be easily generalized to space. In the approach, we can begin by applying fuzzy clustering techniques [3,23,24] to cluster the time series data, and then after that build a fuzzy system that will then form a classification method for the particular problem under consideration.

Assuming that we have *n* objects O_1_, O_2_, …, O_n_, and we can apply fuzzy clustering to obtain n pairs (X_i_, Y_i_) i = 1, n, that are the corresponding centers of the n clusters. Based on these centers a fuzzy system can be established in the following form:

If X is x_1_ and Y is y_1_ then Object is O_1_ If X is x_2_ and Y is y_2_ then Object is O_2_ If X is x_n_ and Y is y_n_ then Object is O_n_ (3)

The fuzzy system of Equation (3) can be applied to classification or time series prediction problems because both cases have the same general structure. The complete design of the fuzzy system in (3) needs to establish the membership functions for all the fuzzy sets of the X and Y linguistic variables.

## 4. Proposed Approach with a Combination of Fractal Dimension and Fuzzy Logic

Let us consider in more detail the time series analysis classification problem. Let the sequence y_1_, y_2_, …, y_n_ represent an arbitrary time series with *n* values. For achieving classification for a time series, the data need to be analyzed and then trends and periodicities of the series can be extracted. If we assume that the time series is clustered into n objects O_1_, O_2_, …, O_n_, then a fuzzy system can be built as outlined in Section 3. In the hybrid approach, the complexity of the objects O_1_, O_2_, …, O_n_ as estimated by their respective fractal dimensions in now considered. To this end, the linear dim_1_ and non-linear dim_2_ fractal dimensions, with fuzzy values x_1_, x_2_, …, x_n_, and y_1_, y_2_, …, y_n_, are used respectively in the fuzzy system. The two different variants of the dimension (linear and non-linear are different in their approach to approximate the data) offer as outputs different numeric values to approximate the dimension and we decided to perform the classification with the two variants to enhance classification accuracy. Then a fuzzy system for time series classification can be outlined in the form indicated below.

If dim_1_ is x_1_ and dim_2_ is y_1_ then classification is O_1_ If dim_1_ is x_2_ and dim_2_ is y_2_ then classification is O_2_ … If dim_1_ is x_n_ and dim_2_ is y_n_ then classification is O_n_ (4)

**Figure 2:**
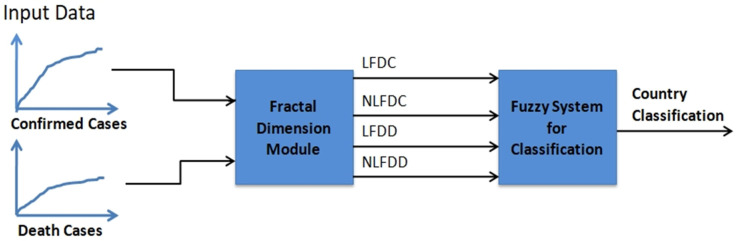
Proposed method with the fractal dimension module and the fuzzy system.

**Figure 3:**
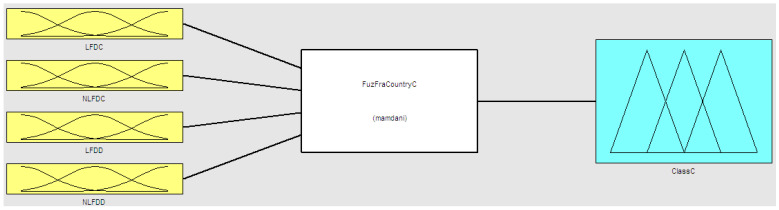
Architecture of the model for Country Classification based on COVID-19 data.

**Figure 4:**
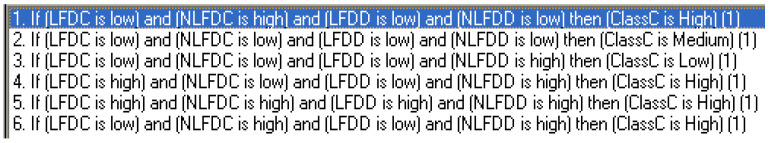
Fuzzy if-then rules expressing the classification knowledge in the fuzzy system.

**Figure 5:**
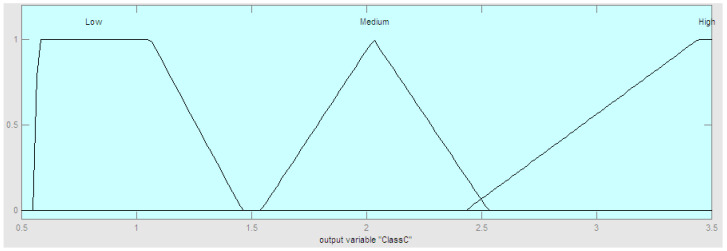
Membership functions of the Output, which is the Classification of Countries.

**Figure 6:**
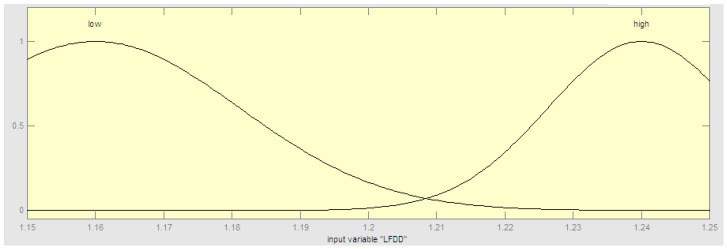
Input membership functions for the input variable, which is the linear fractal dimension of death cases (LFDD).

In Equation (4), we need to establish the membership functions of the two variants of the fractal dimension. This fuzzy system is built with the Mamdani method, and the center of area as the defuzzification approach. In the particular case of country classification based on COVID-19 data, two relevant time series were considered: Confirmed and death data. The main reason for doing this is that both time series provide important and relevant information on the nature of the problem. In summary, based on the above we designed a fuzzy system with four inputs and one output. The four inputs are defined as follows: Linear fractal dimension of confirmed cases (LFDC), nonlinear fractal dimension of confirmed cases (NLFDC), linear fractal dimension of death cases (LFDD), and nonlinear fractal dimension of death cases (NLFDD). In this case, we consider two linguistic values: Low and high, to make a representation of the idea of low and high values of the dimensions. The output linguistic variable is the Class of the Country (ClassC) with three fuzzy values representing the view that countries can be classified with three COVID-19 pandemic emergency levels: High, Medium, and Low. The fuzzy rules were constructed empirically based on previous data and the calculated fractal dimension values. The proposed method is summarized in Figure 2. The method was implemented in software and in the following Figures we illustrate the components of the fuzzy system. First, the structure of the hybrid model is illustrated in Figure 3. The fuzzy system consists of rules to make the classification, which are summarized in Figure 4. The fuzzy rules express our general knowledge for country classification based on the time series complexity. In this case, a higher dimension reflects a higher complexity of the time series. In the output, we use one triangular and two trapezoidal membership functions as depicted in Figure 5. In this case, we decided to use three membership functions as we had the goal to classify countries in three classes (Low, Medium, and High). We illustrate in Figure 6 the membership functions corresponding to only one (for illustration purposes) of the input variables. In this Figure 6, we use two Gaussian functions corresponding to the low and high values, respectively.

The fuzzy rules in the fuzzy model are presented in Figure 4. Each fuzzy rule represents part of the knowledge in classifying the countries according to the complexity of their corresponding time series. This knowledge is based on expert knowledge about classification. We consulted experts on healthcare, medicine, and epidemiology from institutes in Tijuana, Mexico regarding the COVID-19 pandemics and we have related their knowledge with the geometrical form of the times series plots to arrive to the fuzzy rules shown in Figure 4. The general idea is that when the fractal dimension is high the complexity of the time series plot is high and this will indicate a more complex COVID-19 situation in a country. On the other hand, if the fractal dimension tends to be low then the COVID-19 situation with be more stable and under control. Regarding the form and parameters of the membership functions we used a trial and error strategy to find the final form of the membership functions shown in Figure 5 and Figure 6, respectively.

We have to say that we decided to use the specific time window mentioned above to validate the method, but the method can also be used for other time windows and of course with the evolution of the pandemics the classification of a specific country can change depending on what is happening in the new time window. For example, if the plot of the time series is now stable, then in a new time window (the plot is close to linear form) and then the dimension will be close to 1 and the Class of the Country will be Low.

## 5. Experimental Results

The method that uses the fractal dimension in the fuzzy system to build a classification of countries in the world is tested in this section. First a clustering process was performed and then a classification was constructed by considering three classes according to the number of COVID-19 cases.

**Figure 7:**
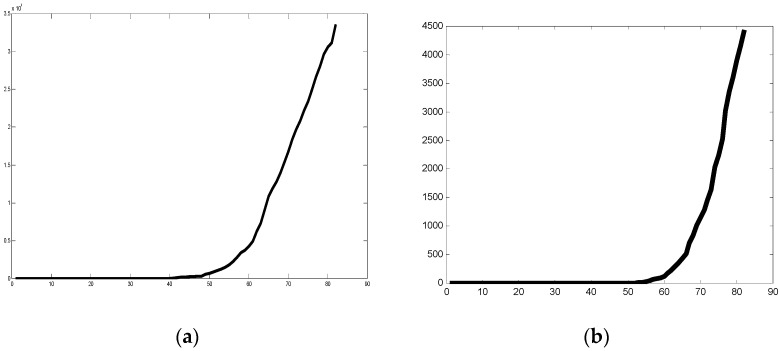
(**a**) Plot of confirmed cases for Belgium, (**b**) plot of death cases for Belgium (2 January 2020 to 31 March 2020).

**Figure 8:**
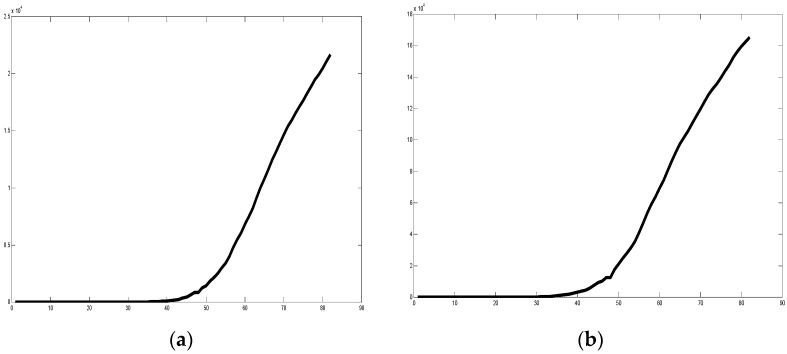
(**a**) Plot of confirmed cases for Italy, (**b**) plot of death cases for Italy (2 January 2020 to 31 March 2020).

**Figure 9:**
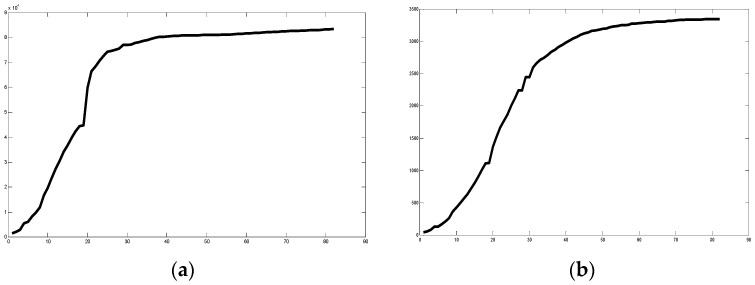
(**a**) Plot of confirmed cases for China, (**b**) plot of death cases for China (2 January 2020 to 31 March 2020).

The database considered for undertaking the experimental work was extracted from the Humanitarian Data Exchange (HDX) [8]. This database is comprised of COVID-19 data from the countries from 2 January 2020 to 31 March 2020. The consulted datasets for achieving the set of experiments and results presented in this section are the following ones: time_series_covid19_confirmed_global, time_series_covid19_recovered_global, and time_series_covid19_deaths_global. The data consist of the confirmed, recovered, and deaths cases of patients from the countries, respectively. In Figure 7, the plot illustrates the trend in the data for Belgium, clearly showing the COVID-19 Confirmed cases for the time period of 2 January 2020 to 31 March 2020 in Figure 7a and then similarly death cases in Figure 7b. In Figure 8a, similar graphical representation for Italy is illustrated for both confirmed and death cases. Figure 9 similarly shows the confirmed and death cases for China. In Figure 7, Figure 8 and Figure 9 the x-axis represents the days in the mentioned interval, so a value of 1 is representing 2 January 2020 and so on.

We can appreciate in the previous Figures the different forms of the graphs, which although similar are different and then their fractal dimensions are also different.

We show in Table 1 and Table 2 the calculations of the fractal dimension for the 11 countries used in the fuzzy fractal model. In Table 1, the fractal dimension for the plots of confirmed cases are presented, including the linear fractal dimension, nonlinear fractal dimension (NL), the correlation for linear (CL), and correlation for nonlinear (CNL). We can note in the table that the values of the linear dimension are always lower that the nonlinear fractal dimension and the correlations of the second one are always closer to 1. We decided to use both types of fractal dimension to have different sources of information or measures of complexity of the countries. Both fractal dimensions are applied as the inputs to the fuzzy system for performing an accurate classification of the countries. Correlation values are closer to 1 in the nonlinear fractal dimension, which is better because the nonlinear regression fits better the data.

In Table 2, the fractal dimension for the plots of death cases are presented, including the linear fractal dimension, nonlinear fractal dimension (NL), the correlation for linear (CL), and correlation for nonlinear (CNL).

**Figure 10:**
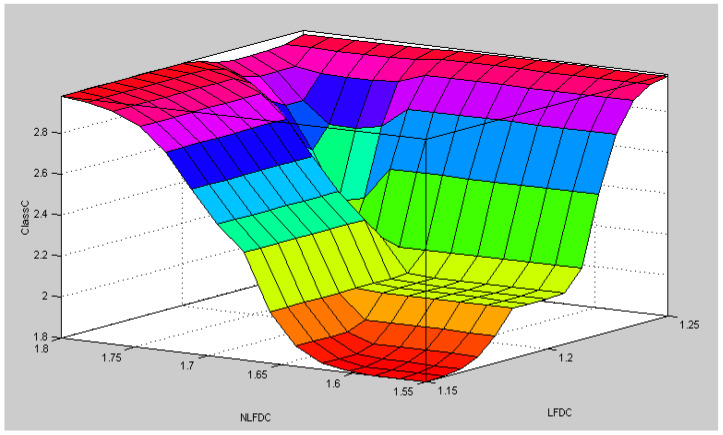
Nonlinear surface definitions for the classes by the fuzzy fractal model.

After the implementation of the fuzzy model represented in Figure 3, we can note that the nonlinear surface of the model is as illustrated in Figure 10, where we can appreciate the nonlinear form in which all the classes are defined. The colors in Figure 10 do not have a particular meaning to be assigned. The colors are just used to help illustrate the form of the surface and this is the usual way in which the fuzzy logic software shows this kind of figure. In the particular case of our developed system, this figure is generated after building the fuzzy system with the fuzzy logic toolbox of the Matlab programming language.

**Figure 11:**
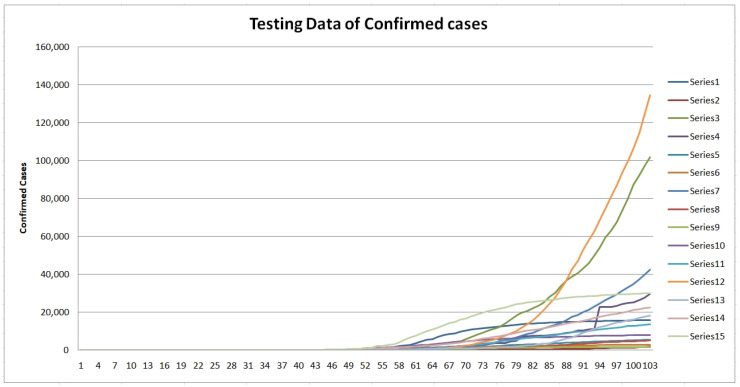
Testing dataset of confirmed cases for the 15 countries (1 April 2020 to 12 July 2020).

**Figure 12:**
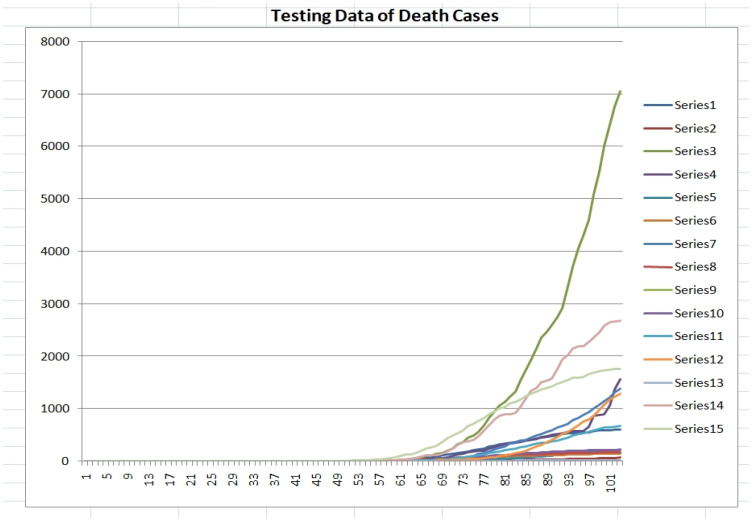
Testing dataset of the death cases for the 15 countries (1 April 2020 to 12 July 2020).

As previously mentioned, we did build the fuzzy fractal model based on the data of the 11 countries in Table 1 and Table 2. Now, with the purpose of validating the model we consider another set of countries (not known to the model) as testing data set. The time series are for the following 15 countries: Austria (series 1), Bolivia (series 2), Brazil (series 3), 4 Ecuador (series 4), Finland (series 5), 6 Greece (series 6), India (series 7), Morocco (series 8), New Zealand (series 9), Norway (series 10), Poland (series 11), Russia (series 12), Singapore (series 13), Sweden (series 14), and Switzerland (series 15). In Figure 11 and Figure 12, the datasets of confirmed and death cases for the 15 countries are illustrated, respectively.

In Table 3, we can find the validation of the proposed method with the data of 15 countries not previously seen by the method. After applying the method with the values of the fractal dimension the fuzzy model produces a numeric output that can be associated with a class. The validation consists on contrasting the predicted class by the method (numeric value) with the real one according to the experts. The experts are deciding on the particular classes of the countries based on the nature of the COVID-19 time series and also other relevant data of the country, like propensity of getting ill due to COVID-19 (due to diabetes or hypertension), classes or neighboring countries, and others. The experts meet and decide by voting on the class of the countries.

We have to say that the fuzzy model output, due to the defuzzification, produces a number, and the assigned class is the closest integer number. In 14 of 15 values, the fuzzy model produces the correct classification. Only in the case of Russia is there one incorrect classification, but the output of 2.45 is close to the threshold of 2.5. The countries that are classified as High, like Bolivia and Finland, have a relative high number of COVID cases and the dynamics of their data is complex and as a consequence they will most probably need to make stronger actions to control the spread of the virus. If in fact these control actions are made, then in a future time window, the fractal dimension will decrease and correspondingly the class will change from High to Medium or Low.

**Figure 13:**
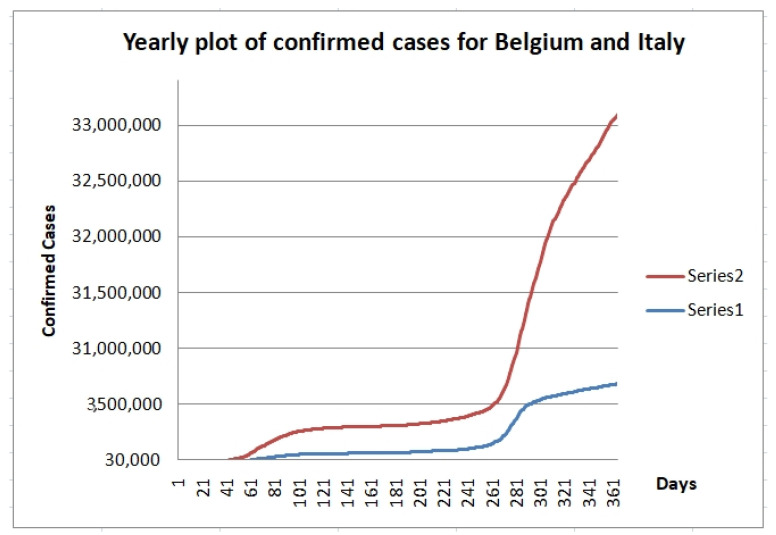
Time series of Belgium and Italy of the 22 January 2020 to 20 January 2021.

We also considered two countries (Belgium and Italy) in two more recent periods in time to illustrate that the fractal dimensions of the time series are changing according to behavior of the pandemic in each country. We selected two periods in time, which we called period 1 (July and August of 2020) and period 2 (November and December of 2020). In Table 4, we can find the values of the fractal dimension for both countries in both time periods, where we can appreciate that their classification of Class 2 is as expected by the experts. Notably, we have to say the classification of both countries in the initial period (January to March of 2020) was of Class 3 because they had higher fractal dimension values on that period. This reflects the fact that both countries had made good a control of the pandemic and for this reason the situation now is not so problematic, although still can be improved to eventually get to Class 1. In addition, we are showing another experiment by considering yearly data of these two countries, in this way analyzing the average dynamics of the COVID-19 time series. We show in Figure 13 the series 1 (Belgium) and series 2 (Italy) for the year data from 22 January of 2020 to 20 January of 2021. The fractal dimensions (linear and nonlinear) of Belgium are 1.093 and 1.586, respectively. The fractal dimensions of Italy are 1.093 and 1.587, which are very similar to the ones of Belgium. Actually, both countries have behaved similarly as can be appreciated in Figure 13. Both countries are in Class 2 based on the yearly values of fractal dimensions. However, we can note from Figure 13, that there are three different time windows: Initial rising period, then stable horizontal period and the again rising period, so the class is changing with time.

**Figure 14:**
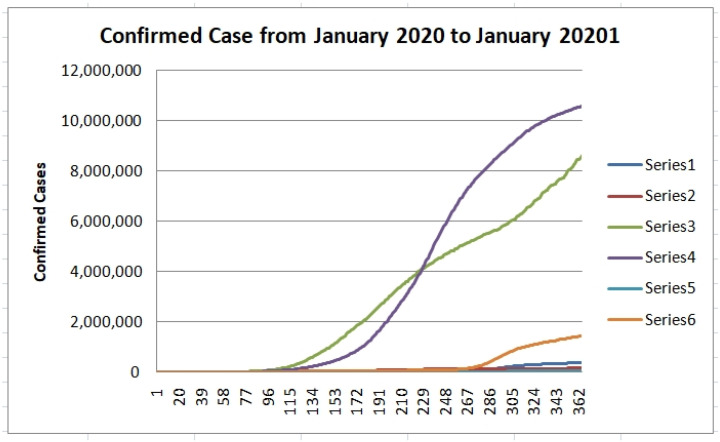
Time series of six countries of the 22 January 2020 to 19 January 2021.

We also show in Figure 14 the plots of the confirmed cases from several countries for a period of almost one year (22 January 2020 to 19 January 2021) to illustrate the dynamic behavior of the pandemics in a longer time window. In Figure 14, we can appreciate six time series of different countries (Series 1 is of Austria, Series 2 is of Bolivia, Series 3 is of Brazil, Series 4 is from India, Series 5 is Norway, and Series 6 is Poland). We can appreciate that India has more confirmed cases, followed by Brazil and then Poland.

## 6. Applications of the Proposed Classification Method

We describe in this section the applicability of the proposed classification method in different areas of application, like time series prediction, control, and modeling spatial-temporal evolution of the pandemics. However, we will illustrate this with the particular application of time series prediction to show that the classification approach helps in providing a good prediction for the COVID-19 time series. We have to say that the classification approach presented in this paper is only based on temporal information, but we can also use spatial information and the classification in that case can be based on clustering techniques applied over the spatial data. This is another important facet of the problem, as COVID-19 is also evolving on space, not only in time. We plan to work on this spatial temporal approach by combining the fuzzy fractal method with self-organizing maps in the near future. Another possible application of the fuzzy fractal approach is in control, which means performing intervention control actions depending on the actual class of the country, which means the size of the control actions will depend on the class, in other words a country classed as High will need higher control actions. We have to remember that the class of the country is not a static value as it depends on evaluation of the complexity on particular window in time, so after initial control actions in the next window, the Class value can go down if the control action was adequate. Based on preliminary studies we have found that in some cases with additional one-month data we are able to detect a change in the class of the country with the proposed approach, but this is for the cases with higher changes in the time series curve. In other cases, this could require two to three months for a change. This is another interesting area of research that we also want to explore in the near future.

**Figure 15:**
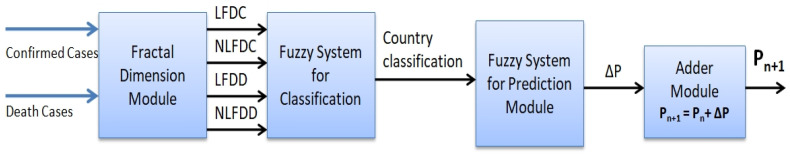
Structure of the approach for time series prediction.

In the particular case of time series prediction, the classification can serve as the basis for establishing the fuzzy rules for prediction. In Figure 15, we illustrate how the approach for time series prediction based on the classification approach is structured. In this Figure, we can appreciate that the first two blocks from left to right are the same as for the classification method, but now the difference is that the classification information is used as the input to a new fuzzy system (block 3 from left to right) that is in charge of making the prediction. This block will produce as an output a numeric value (after defuzzification), which is viewed as an increment to the previous value of the time series and then is added (this is in the Adder Module) to obtain the new predicted value.

The knowledge in the fuzzy system for prediction in this case is that if a country is from the Low Class then the change in the predicted values will be also low (fuzzy value), if the country has a Medium Class then the change will be also medium, and if the Class is high then the change will be high. Then the fuzzy rules for prediction are:

IF Class of Country is *Low* then Increment in Prediction is *Low*
IF Class of Country is *Medium* then Increment in Prediction is *Medium*
IF Class of Country is *High* then Increment in Prediction is *High*

**Figure 16:**
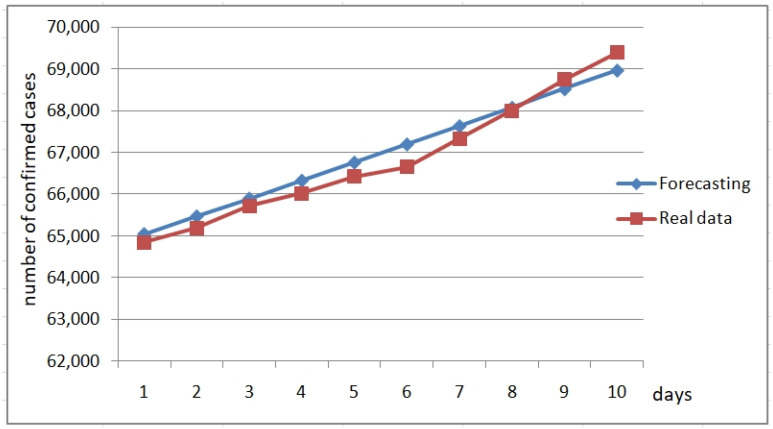
Forecasting the confirmed cases of Belgium from 22 July to 1 August 2020.

**Figure 17:**
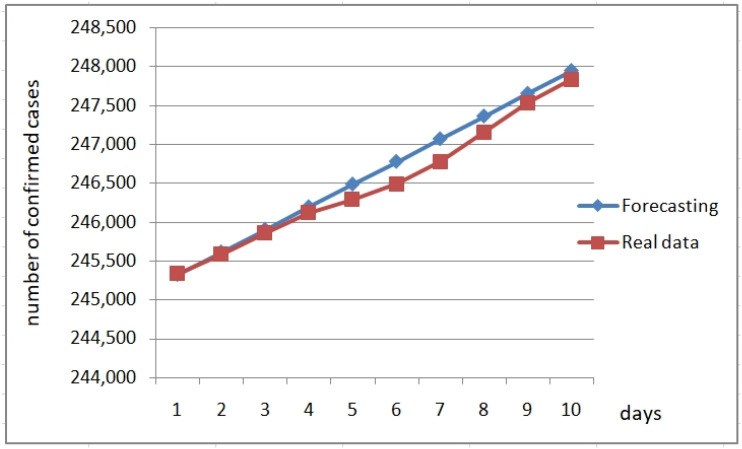
Forecasting Italy confirmed cases from 22 July to 1 August of 2020.

The previous fuzzy rules constitute a forecasting fuzzy model that uses the fuzzy system to obtain the change in the value of the variable. In the following Figures, we show plots of forecasting with the fuzzy fractal approach of these three fuzzy rules for several countries for a more recent period. We are forecasting 10 days ahead (22 July 2020 to 1 August 2020) based on data used for designing the fuzzy model (22 January 2020 to 15 April 2020). Figure 16 illustrates the forecast of confirmed cases for Belgium, where we can appreciate that the forecasted values are very close to the real values. Figure 17 illustrates the forecast of the confirmed cases for Italy. In both cases, the forecasts are close to the real values, which confirms that the fuzzy fractal approach works well in time series prediction.

## 7. Conclusions

We have presented in this work a hybrid intelligent method for classification of countries based on the COVID-19 time series complexity using a prudent mixture of fractal theory and fuzzy logic concepts. Two approximations of the fractal dimension (linear and nonlinear) were applied to estimate the complexity of the dynamics in the time series of the countries. Fuzzy Logic was employed to represent the inherent decision-making uncertainty in performing the classification. The proposed method is formed by a fuzzy model, comprising fuzzy rules, that considers the fractal dimensions as input values and produces as outputs the classification of the countries based on the COVID-19 data. The main contribution of this article is the proposed method hybridizing in a prudent fashion the fractal dimension and fuzzy logic theoretical constructs for realizing an accurate classification of COVID-19 data. Publicly available datasets of 11 countries worldwide have been utilized to construct the fuzzy model with fractal dimensions as fuzzy variables, and 15 different countries were used to validate the effectiveness of the approach. We plan to consider testing the classification approach with more classes to verify if this will result in an improved classification and also the corresponding resulting actions or decisions based on the classes could improve (for example in forecasting, control, and others). We imagine as future work the application of the proposed method in similar problems, as well as elevating the level of fuzzy logic to type-2, which in theory could provide a better representation of the uncertainty in the decision-making process required for achieving a good classification. In addition, we plan to consider the relation of this paper to current works on other facets of the COVID-19 problem, like the ones presented in [25,26,27], or in forecasting the COVID-19 time series [28,29,30]. Finally, we plan to combine our approach with neural network models (like self-organizing maps or ensemble models) to study spatial and temporal patterns of countries, like in [31,32].

## Figures and Tables

**Table 1:** Fractal dimensions for confirmed cases in the countries.

Metric	Fractal Dimension Country Confirmed Cases										
	Belgium	China	France	Germany	Iran	Italy	Mexico	Spain	Turkey	UK	US
Linear	1.1860	1.2210	1.1900	1.2020	1.1910	1.1940	1.1970	1.1860	1.2040	1.2070	1.2040
NL	1.7480	1.7240	1.7440	1.6150	1.7210	1.7220	1.6190	1.7750	1.6080	1.624	1.5930
CL	0.8760	0.8397	0.8369	0.8555	0.8384	0.8449	0.8489	0.8093	0.8446	0.8467	0.8736
CNL	0.9991	0.9949	0.9928	0.9967	0.9927	0.9996	0.9999	0.9944	0.9979	0.9999	0.9980

Linear = box counting linear logarithmic, NL = box counting Nonlinear logarithmic, CL = Correlation Coefficient of Linear calculation, CNL = Correlation Coefficient of Non-Linear calculation.

**Table 2:** Fractal dimensions for death cases in the countries.

Metric	Fractal Dimension Country Death Cases										
	Belgium	China	France	Germany	Iran	Italy	Mexico	Spain	Turkey	UK	US
Linear	1.2080	1.2120	1.1900	1.1780	1.2040	1.1890	1.1780	1.1810	1.2020	1.2120	1.1870
NL	1.6040	1.7190	1.7880	1.7100	1.6230	1.6140	1.8250	1.7890	1.5960	1.6010	1.804
CL	0.8603	0.8356	0.8011	0.8192	0.8948	0.8525	0.7870	0.8097	0.8490	0.8927	0.7942
CNL	0.9978	0.9998	0.9945	0.9958	0.9991	0.9999	0.9944	0.9939	0.9980	0.9998	0.9998

Linear = box counting linear logarithmic, NL = box counting Nonlinear logarithmic, CL = Correlation Coefficient of Linear calculation, CNL = Correlation Coefficient of Non-Linear calculation.

**Table 3:** Validation of the Fuzzy Fractal Classification Method with 15 countries.

Country	LFDC	NLFDC	LFDD	NLFDD	Class	Val Class	Validation
Austria	1.2110	1.6370	1.2080	1.6130	2 (Medium)	2.24	Correct
Bolivia	1.1970	1.7950	1.2020	1.6010	3 (High)	2.99	Correct
Brazil	1.2290	1.6110	1.2110	1.5940	2 (Medium)	2.34	Correct
Ecuador	1.1950	1.6410	1.1990	1.7650	2 (Medium)	1.76	Correct
Finland	1.2350	1.6200	1.1910	1.7610	3 (High)	3.01	Correct
Greece	1.2340	1.7360	1.1990	1.6190	2 (Medium)	2.31	Correct
India	1.2080	1.6270	1.2090	1.6150	2 (Medium)	2.12	Correct
Morocco	1.1850	1.7760	1.2040	1.7460	3 (High)	2.98	Correct
New Zealand	1.1990	1.7840	1.2100	1.7710	3 (High)	2.98	Correct
Norway	1.1990	1.6380	1.2390	1.6270	2 (Medium)	2.16	Correct
Poland	1.1960	1.7310	1.1880	1.7890	3 (High)	2.89	Correct
Russia	1.1950	1.7650	1.2000	1.6070	3 (High)	2.45	Incorrect
Singapore	1.1740	1.7650	1.1750	1.7780	3 (High)	3.03	Correct
Sweden	1.1650	1.5940	1.1760	1.6140	2 (Medium)	2.03	Correct
Switzerland	1.2030	1.7350	1.2070	1.6200	3 (High)	2.91	Correct

**Table 4:** Validation of the Fuzzy Fractal Classification Method for Belgium and Italy.

Country	LFDC	NLFDC	LFDD	NLFDD	Class	Val. Class	Validation
Belgium period 1	1.083	1.594	1.156	1.586	2	2.04	Correct
Belgium period 2	1.089	1.569	1.090	1.573	2	2.03	Correct
Italy period 1	1.101	1.574	1.080	1.587	2	2.03	Correct
Italy period 2	1.083	1.557	1.094	1.561	2	2.03	Correct

## Data Availability

Not applicable.
